# Expression of Concern: A modified fractional short circuit current MPPT and multicellular converter for improving power quality and efficiency in PV chain

**DOI:** 10.1371/journal.pone.0351365

**Published:** 2026-06-09

**Authors:** The PLOS One Editors

The *PLOS One* Editors issue this Expression of Concern for this article [1] because of concerns identified about the peer review process. Readers are advised to interpret the article [1] with caution.
