# Supplementary figures and images for: A modified fractional short circuit current MPPT and multicellular converter for improving power quality and efficiency in PV chain
Source: PLoS One. 2024 Sep 3;19(9):e0309460. doi: 10.1371/journal.pone.0309460 (PMC11371253; doi:10.1371/journal.pone.0309460)

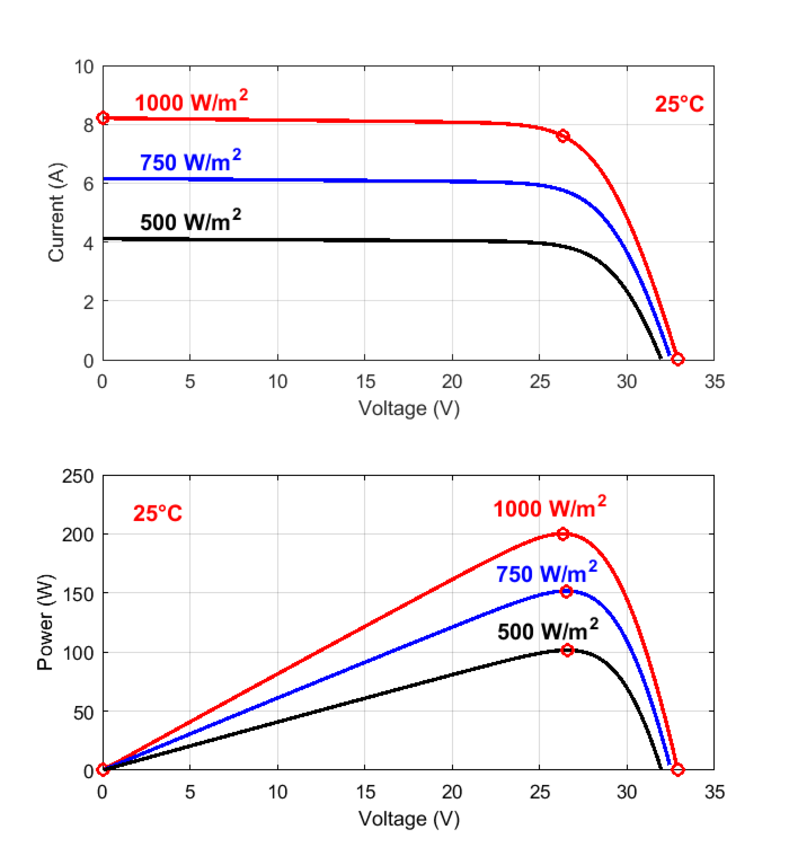

Supplement: S1 Fig — (TIF) [file pone.0309460.s001.tif]

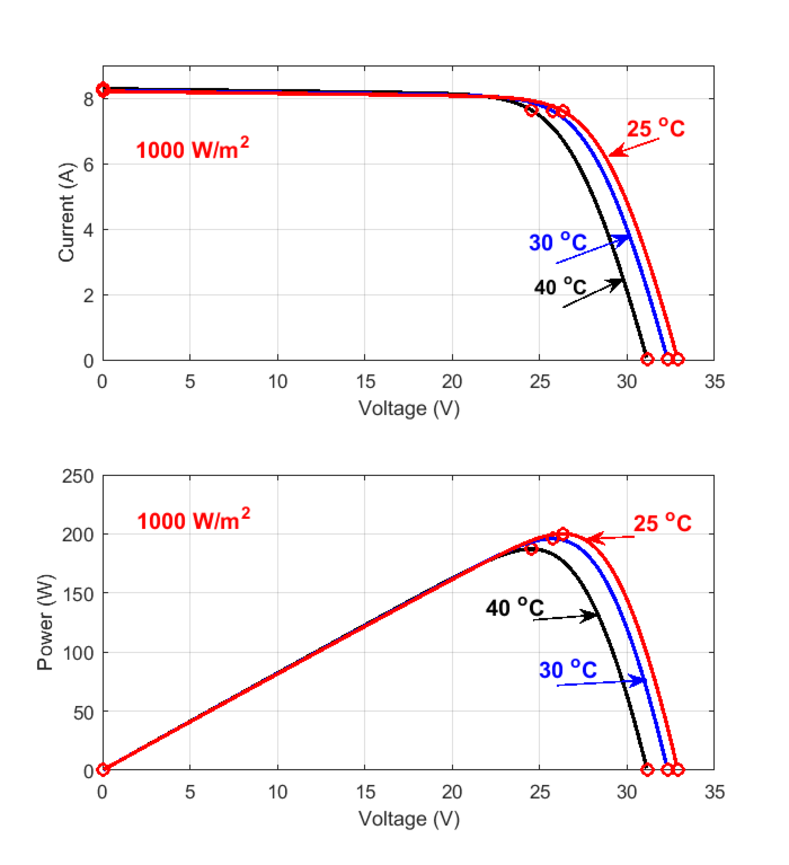

Supplement: S2 Fig — (TIF) [file pone.0309460.s002.tif]

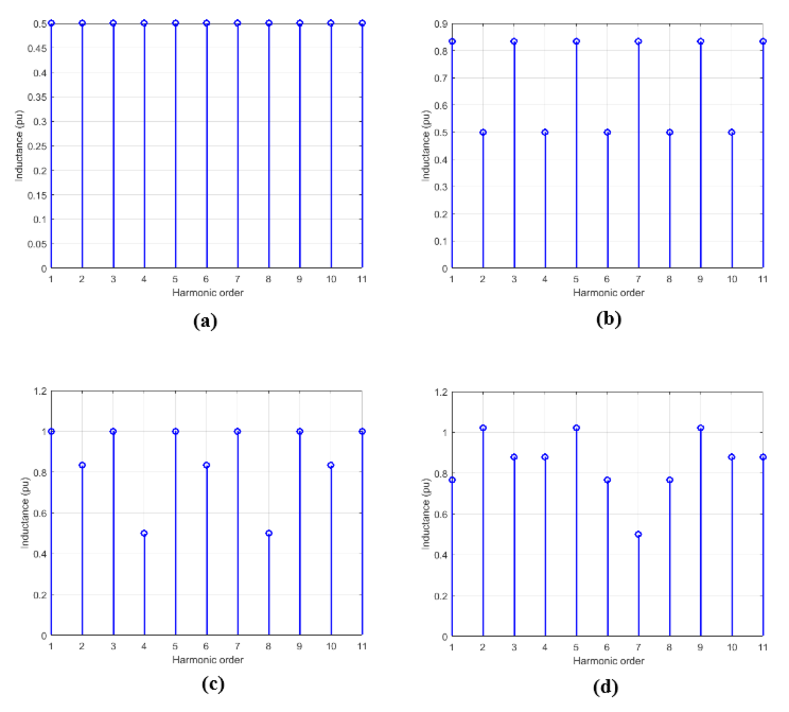

Supplement: S3 Fig — (a) one cell. (b) 2 cells. (c) 4 cells. (d) 7 cells. (TIF) [file pone.0309460.s003.tif]
