# Supplementary material for: A modified fractional short circuit current MPPT and multicellular converter for improving power quality and efficiency in PV chain
Source: PLoS One. 2024 Sep 3;19(9):e0309460. doi: 10.1371/journal.pone.0309460 (PMC11371253; doi:10.1371/journal.pone.0309460)
Supplement: S1 Table — (DOCX) [file pone.0309460.s004.docx]

**S1 Table. Characteristics of Kyocera Solar KC200GT photovoltaic module at constant T= 25°C.**

| **Number of connected strings in parallel : 1** | | | | | |
| --- | --- | --- | --- | --- | --- |
| **Module per string connected in series :1** | | | | | |
| **Short circuit current temperature coefficient**$\boldsymbol{\alpha=0,06\%/^{\circ}C}$ | | | | | |
| **Température** $\boldsymbol{T=25^{\circ}C}$ | | | | | |
| $\boldsymbol{G}$ | $\boldsymbol{V}_{\boldsymbol{OC}}$ | $\boldsymbol{I}_{\boldsymbol{SC}}$ | $\boldsymbol{V}_{\boldsymbol{MPP}}$ | $\boldsymbol{I}_{\boldsymbol{MPP}}$ | $\boldsymbol{P}_{\boldsymbol{MPP}}$ |
| $1000W/m^{2}$ | $32.9V$ | $8.21A$ | $26.3V$ | $7.61A$ | $200.14W$ |
| $750W/m^{2}$ | $32.43V$ | $6.16A$ | $26.52V$ | $5.72A$ | $151.7W$ |
| $500W/m^{2}$ | $31.94V$ | $4.11A$ | $26.58V$ | $3.82A$ | $101.6W$ |
