# Supplementary material for: A modified fractional short circuit current MPPT and multicellular converter for improving power quality and efficiency in PV chain
Source: PLoS One. 2024 Sep 3;19(9):e0309460. doi: 10.1371/journal.pone.0309460 (PMC11371253; doi:10.1371/journal.pone.0309460)
Supplement: S2 Table — (DOCX) [file pone.0309460.s005.docx]

**S2 Table. Characteristics of Kyocera Solar KC200GT photovoltaic module at constant** $\boldsymbol{G=} \boldsymbol{1000}\boldsymbol{W}\boldsymbol{/}\boldsymbol{m}^{\boldsymbol{2}}$ **.**

| **Number of connected strings in parallel : 1** | | | | | |
| --- | --- | --- | --- | --- | --- |
| **Module per string connected in series :1** | | | | | |
| **Short circuit current temperature coefficient**$\boldsymbol{\alpha=0,06\%/^{\circ}C}$ | | | | | |
| **Irradiance** $\boldsymbol{G=1000}\boldsymbol{W/}\boldsymbol{m}^{\boldsymbol{2}}$ | | | | | |
| $\boldsymbol{T}$ | $\boldsymbol{V}_{\boldsymbol{OC}}$ | $\boldsymbol{I}_{\boldsymbol{SC}}$ | $\boldsymbol{V}_{\boldsymbol{MPP}}$ | $\boldsymbol{I}_{\boldsymbol{MPP}}$ | $\boldsymbol{P}_{\boldsymbol{MPP}}$ |
| $25^{\circ}C$ | $32.9V$ | $8.21A$ | $26.3V$ | $7.61A$ | $200.14W$ |
| $30^{\circ}C$ | $32.32V$ | $8.235A$ | $25.74V$ | $7.609A$ | $195.8W$ |
| $40^{\circ}C$ | $31.15V$ | $8.284A$ | $24.55V$ | $7.624A$ | $187.1W$ |
