# Supplementary material for: A modified fractional short circuit current MPPT and multicellular converter for improving power quality and efficiency in PV chain
Source: PLoS One. 2024 Sep 3;19(9):e0309460. doi: 10.1371/journal.pone.0309460 (PMC11371253; doi:10.1371/journal.pone.0309460)
Supplement: Attachment — Submitted filename: Comments to the Author_PLOS.docx [file pone.0309460.s006.docx]

**Comments to the Author:**

The manuscript is well presented with use of multicellular converter in PV chain for improving efficiency and to reduce power oscillations. The paper can be accepted with minor revision and grammatical corrections. The following points can be followed to update/strengthen the manuscript:

1. The manuscript has some grammatical mistakes - Please check it.
2. Literature review is shallow
3. Authors mentioned thaat the efficiency is 99.65% for Inductance current and 99.6% for sliding method which is not convincing due to the passive components involved in the boost converter.
4. The analysis of traditional DC/DC converters can be presented with respect to various duty cycle with the suggested MPP.
5. Novelty of the proposed work should be established by comparing the same with comparable work. Authors can justify or compare the following MPPT based boost converter and justify how the presented MPP technique is superior to other MPPT approaches.
i. Nagaraja Rao, S., Anisetty, S. K., Manjunatha, B. M., Kiran Kumar, B. M., Praveen Kumar, V., & Pranupa, S. (2022). Interleaved high-gain boost converter powered by solar energy using hybrid-based MPP tracking technique. *Clean Energy*, *6*(3), 460-475.
ii. Veerabhadra, & Nagaraja Rao, S. (2022). Assessment of high-gain quadratic boost converter with hybrid-based maximum power point tracking technique for solar photovoltaic systems. *Clean Energy*, *6*(4), 632-645.
6. In the abstract and conclusion, the results performances should be reflected which helps to improve the quality of the manuscript. Add the results values in the abstract.
